# Effects of Heat Treatment on Physicochemical Properties of *Moringa oleifera* Lam. Leaf Protein

**DOI:** 10.3390/ijms26041647

**Published:** 2025-02-14

**Authors:** Chuyu Xi, Wenjie Li, Zhiguo Xu, Jing Xie, Xiaoyu Gao, Dan Feng, Yang Tian, Shuang Song

**Affiliations:** 1 College of Food Science and Technology, Yunnan Agricultural University, Kunming 650201, China; xichuyu@stu.ynau.edu.cn (C.X.); liwenjie@stu.ynau.edu.cn (W.L.); 2023240101@stu.ynau.edu.cn (Z.X.); jingxie0624@163.com (J.X.); 2018014@ynau.edu.cn (X.G.); fengdan@stu.ynau.edu.cn (D.F.); 2 Yunnan Key Laboratory of Precision Nutrition and Personalized Food Manufacturing, Yunnan Agricultural University, Kunming 650201, China; 3 Engineering Research Center of Development and Utilization of Food and Drug Homologous Resources, Ministry of Education, Yunnan Agricultural University, Kunming 650201, China

**Keywords:** *M. oleifera* leaves, food allergen, ammonium sulfate precipitation, heat treatment

## Abstract

*M. oleifera* leaves represent a novel and nutritious food. Prior research has demonstrated that *M. oleifera* leaves can elicit allergic responses in BALB/c mice. Based on these findings, further studies were conducted to investigate the effects of heat treatment on the allergenicity, particle size, zeta potential, total sulfhydryl (TSH) content, hydrophilicity and hydrophobicity, ultraviolet spectrum, and intrinsic fluorescence spectrum of *M. oleifera* leaf protein. Additionally, in vitro digestion experiments were carried out to gain further insights into the protein’s behavior under these conditions. The experiment simulated the alterations in *M. oleifera* leaf protein during the processes of cooking and digestion. The findings of this experiment can provide certain guidance for the processing of *M. oleifera* leaf products. The hydrophilicity, hydrophobicity, transmembrane region, antigen index, calcium binding site, spatial structure, and homology of *M. oleifera* leaf fructose 1,6 bisphosphate aldolase (FBA) were simulated and calculated based on the amino acid sequence of the 36 kDa allergen. These parameters collectively serve to indicate the allergenic activity of the peptide. The findings of the analysis align with the outcomes of the sensitization experiments, suggesting that the FBA of *M. oleifera* leaves is indeed consistent. In conjunction with the heat treatment experiments, this research can inform the preparation of *M. oleifera* leaf foods and provide a foundation for further investigation into *M. oleifera* leaf allergens.

## 1. Introduction

*Moringa oleifera* Lam., or drumstick tree (*M. oleifera*), is the most widely distributed and cultivated member of the *Moringaceae* family. *M. oleifera* is indigenous to India, where it is cultivated as a vegetable [1]. *M. oleifera* exhibits traits associated with cold hardiness, ease of cultivation, and high yield. *M. oleifera* is cultivated on a global scale as a cost-effective, abundant, and sustainable source of protein, as well as a food supplement suitable for human consumption [2]. A substantial body of research has demonstrated that the leaves, flowers, fruits, seeds, seed oil, bark, and roots of *M. oleifera* possess nutritional or medicinal value. The data demonstrate that *M. oleifera* and its extracts have a range of biological activities, including antibacterial, anti-tumor, antioxidant, anti-inflammatory, and immunomodulatory. A number of active constituents have been identified, including multiple alkaloids and sterols, polyphenols and phenolic acids, fatty acids, flavanoids and flavanol glycosides, glucosinolates and isothiocyanates, terpenes, and anthocyanins [3]. In China, *M. oleifera* leaf has been listed as a “New Resource Food”, and the *M. oleifera* industry has been the subject of considerable development and promotion efforts [4].

Nevertheless, there have been isolated reports of allergic reactions to *M. oleifera* leaf in individuals exposed to *M. oleifera* products. There have been reports indicating that *M. oleifera* seeds may be a potential occupational asthma trigger for cosmetic line workers [5]. *M. oleifera* leaf, the leafy part of the same plant, is also a potential food allergen; however, there is a paucity of reliable clinical data and epidemiologic studies on *M. oleifera* leaf allergies. Food allergies are a growing public health problem worldwide and its prevalence is increasing every year. Despite the potentially fatal consequences of a food allergy, there is currently no cure for the disorder. Instead, management is limited to the avoidance of allergens or the treatment of symptoms [6]. It is, therefore, crucial to purify and identify allergens to develop effective interventions for food allergies. In recent years, a limited number of reports have emerged concerning *M. oleifera* leaf allergies. It has been demonstrated that *M. oleifera* leaf protein can induce sensitization in BALB/c mice [7]. Furthermore, proteomic and in silico studies indicated that the *M. oleifera* leaf may contain potential allergens: morintides and nsLTP [8]. Nevertheless, there is currently no published research on the purification of *M. oleifera* leaf allergens.

In a previous study, we demonstrated that *M. oleifera* leaf protein exhibits blood coagulation activity, indicating that *M. oleifera* leaf protein contains lectins. The term “lectin” is used to describe a class of non-immune proteins that have the ability to bind specific sugars. These proteins are capable of reversibly binding to one or more proteins that can bind reversibly to monosaccharides or oligosaccharides without altering their structure [9]. Lectins are widely distributed in various vegetative organs of plants and regulate the physiological functions of these organism, for example, stress resistance [10]. It is hypothesized that certain lectins may serve as cancer markers and could be used to treat cancer [11]. It has been reported that a water-soluble lectin (WSMoL) is present in *M. oleifera* seeds [12]. WSMolis a type of water purification coagulant protein [13] that has been demonstrated to possess antibacterial [14,15], antioxidant [12], and anti-inflammatory [16] activities. It is important to note, however, that the use of high concentrations of WSMol may pose a risk to human health [17]. In particular, a number of food plant lectins have been identified as potential food allergens, including legume lectins, wheat germ agglutinin (WGA), nictaba-related lectins, phytohemagglutinin (PHA), etc. [18]. However, the majority of studies examining the sensitizing activity of dietary lectins remain inconclusive. This may be attributed to the non-specific binding of dietary lectins to the carbohydrate moiety of IgE antibodies, which hinders the accurate assessment of their allergenic potential [19].

The most employed methods for food desensitization encompass thermal physical processing, non-thermal physical processing, chemical modification, and biological treatments. Heat treatment is a straightforward processing method that has been demonstrated to be an effective means of reducing the potential allergenicity of foodstuffs. It has been demonstrated that heat treatment can desensitize certain food allergens, such as elderberry and kidney bean lectins [20,21].

In this study, the *M. oleifera* leaf allergen was extracted from fresh *M. oleifera* leaves. The three proteins with the highest contents were 56 kDa for chymosin, 36 kDa for FBA, and a 23 kDa band formed by its cleavage. To investigate the thermal stability of the *M. oleifera* leaf allergen, the protein was subjected to heat treatment (simulated stewing), and the subsequent changes in its hemagglutination activity were examined. The impact of heat treatment on the physicochemical and structural characteristics of the *M. oleifera* leaf allergen was evaluated through alterations in the particle size and zeta potential, total sulfhydryl content, solubility, and surface hydrophobicity, in addition to modifications in the ultraviolet spectrum (UV spectrum), intrinsic fluorescence spectrum, and degree of aggregation. Pepsin and trypsin were employed to simulate the in vitro digestion of the *M. oleifera* leaf allergen, and the resulting digestibility was quantified. Subsequently, the function and structure of the *M. oleifera* leaf allergen were predicted using bioinformatics.

## 2. Results

### 2.1. M. oleifera Leaf Allergen Extraction and Sensitizing Activity Determination

**Figure 1:**
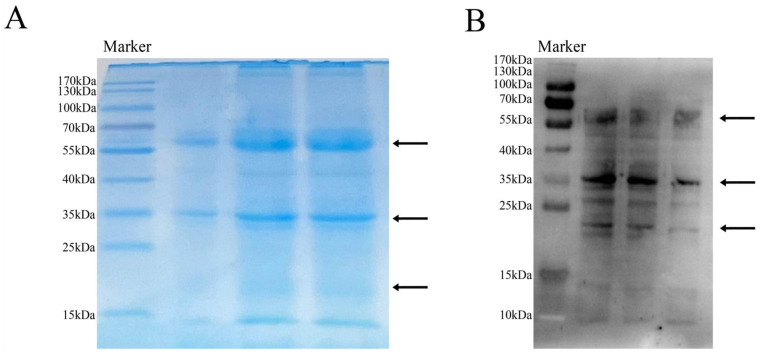
*M. oleifera* leaf allergen extraction and sensitizing activity determination. (**A**) SDS-PAGE of *M. oleifera* leaf allergen; (**B**) allergenic activity of *M. oleifera* leaf allergen. The two image samples are both derived from *M. oleifera* leaf protein, and the arrows in (**A**) indicate relatively concentrated bands. The arrow in (**B**) indicates the bands with a higher degree of IgE binding.

The extracted *M. oleifera* leaf allergen was analyzed using SDS-PAGE, as shown in Figure 1A. The protein with a molecular weight of 60 kDa was higher, and the molecular weight bands of 56, 45, 36, and 23 kDa were deeper. The 56 kDa protein has been identified as *Moringa* leaf rennet [22]. Our experiment found that after the purification of the 36 kDa protein, it gradually degrades to produce a 23 kDa band.

The allergic activity of the *M. oleifera* leaf allergen was determined using immunoblotting, as shown in Figure 1B. The results showed that the *M. oleifera* leaf allergen could specifically bind IgE, and there was more binding at 23, 26, and 36 kDa, which proved that these proteins had allergic activity. Among them, the binding strength between the 36 kDa protein and mouse IgE is relatively higher, so we have identified it as the main peptide in the *M. oleifera* leaf allergen. Based on SDS-PAGE images, we believe that apart from the main bands we labeled, all other signals binding to IgE are generated by the degradation of three major proteins.

### 2.2. Determination of Center Temperature in M. oleifera Leaf Allergen

**Figure 2:**
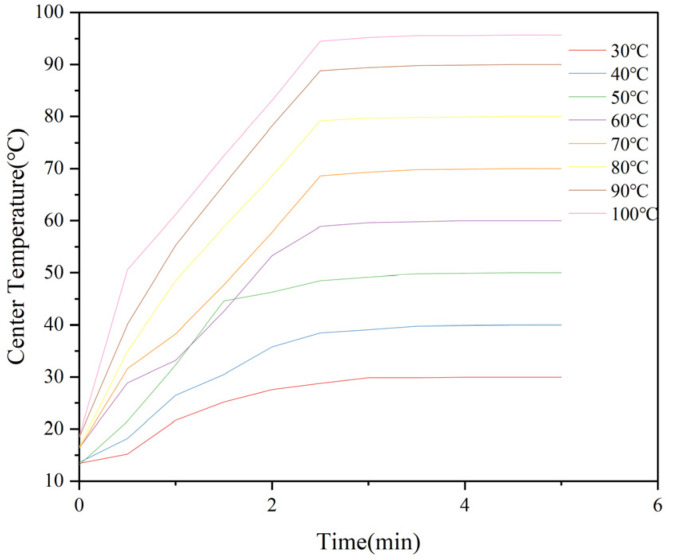
The center temperature of the *M. oleifera* leaf allergen solution during heat treatment.

The time–temperature curve measured during the heat treatment of the *M. oleifera* leaf allergen center is shown in Figure 2. The center temperature of all specimens increased rapidly and finally reached the target temperature of the heat treatment. Due to the high altitude of the laboratory, the maximum temperature of the sample center is 95.4 °C. In the following experiments, 95.4 °C was used as the highest temperature for heat treatment. We aim to reach the specified temperature within 4–5 min, so that the reaction time is the same at the specified temperature, which can help us avoid errors caused by different heating times.

### 2.3. SDS-PAGE of M. oleifera Leaf Allergen After Heat Treatment

**Figure 3:**
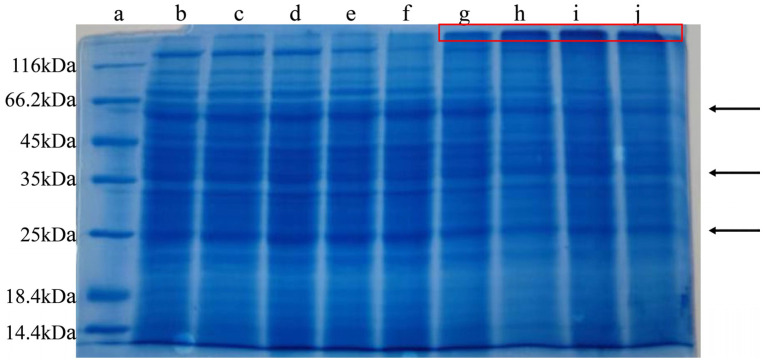
SDS-PAGE of *M. oleifera* leaf allergen after heat treatment. a: marker; b: control; c: 30 °C; d: 40 °C; e: 50 °C; f: 60 °C; g: 70 °C; h: 80 °C; i: 90 °C; j: 100 °C. The arrow indicates the main protein in *Moringa* leaves. The color of some bands in the red box is deepened, which indicates that protein aggregation has occurred.

The effect of the heat treatment on protein aggregation in the *M. oleifera* leaf allergen was analyzed using SDS-PAGE. As shown in Figure 3, the molecular weight distribution of the *M. oleifera* leaf allergen is relatively broad, and the bands are continuous and fuzzy. The continuous banding may be because the *M. oleifera* leaf allergen is composed of many proteins with similar molecular weights or due to the breakdown of key proteins. After heat treatment, the band strength decreased in the range of 60–120 kDa. In addition, the intensity in the band larger than 120 kDa increased with a rising temperature. The SDS-PAGE results showed that the denaturation of proteins during heating may result in the aggregation of proteins and the formation of proteins with higher molecular weights.

### 2.4. Effect of Heat Treatment on Hemagglutination Activity of M. oleifera Leaf Allergen

The effect of the heat treatment on the hemagglutination activity of the *M. oleifera* leaf allergen was analyzed using rabbit blood. As shown in Table 1, when the treatment temperature is below 60 °C, the *M. oleifera* leaf allergen is stable and has strong hemagglutination activity. With the increase in temperature, the hemagglutination activity of the *M. oleifera* leaf allergen gradually decreases until 90 °C, when its hemagglutination activity is completely lost.

### 2.5. Effect of Heat Treatment on Sensitizing Activity of M. oleifera Leaf Allergen

**Figure 4:**
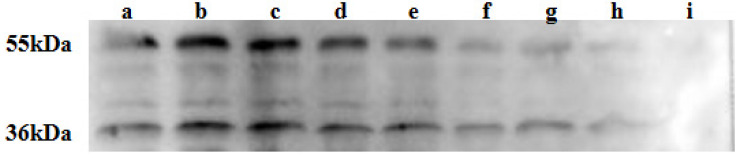
Effect of heat treatment on sensitizing activity of *M. oleifera* leaf allergen. a: control; b: 30 °C; c: 40 °C; d: 50 °C; e: 60 °C; f: 70 °C; g: 80 °C; h: 90 °C; i: 100 °C. The two bands illustrated in the figure are examples of strong IgE-binding bands extracted from *M. oleifera* leaves. As illustrated in the figure, the bands exhibit a gradual weakening and a concomitant decrease in the IgE binding ability with the enhancement of the heat treatment.

The influence of the heat treatment on the sensitizing activity of the *M. oleifera* leaf allergen was analyzed using a Western blot. As shown in Figure 4, after the heat treatment, the allergic activity of *M. oleifera* leaf allergen at 36 and 55 kDa decreased with an increasing temperature, and there was almost no sensitizing activity at 100 °C, indicating that the *M. oleifera* leaf allergen lost its sensitizing activity after the heat treatment.

### 2.6. Analysis of M. oleifera Leaf Allergen Denaturation Temperature

**Figure 5:**
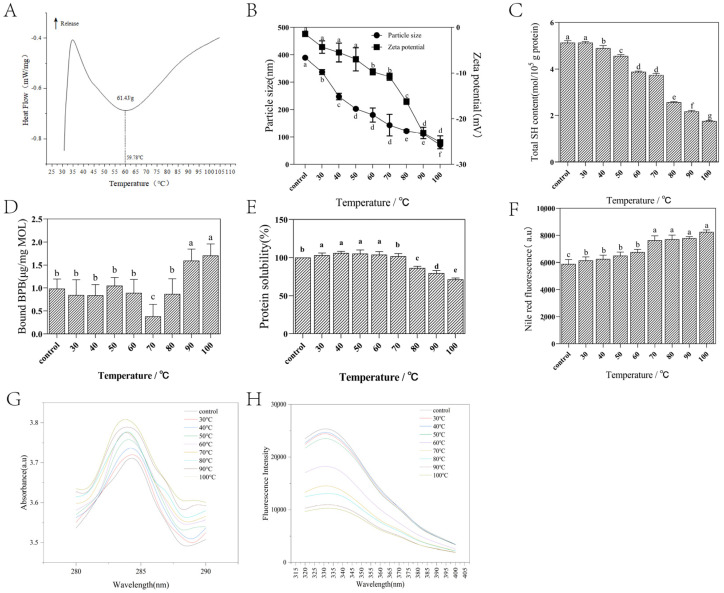
(**A**) Denaturation temperature of *M. oleifera* leaf allergen. (**B**) Effect of heating temperature on particle size and zeta potential of *M. oleifera* leaf allergen. (**C**) Effect of heating temperature on total sulfhydryl content of *M. oleifera* leaf allergen. (**D**) Effect of heating temperature on surface hydrophobicity of *M. oleifera* leaf allergen. (**E**) Effect of heating temperature on the solubility of the *M. oleifera* leaf allergen. (**F**) Effect of heating temperature on protein aggregates of *M. oleifera* leaf allergen. (**G**) Effect of heat treatment on UV spectrum of *M. oleifera* leaf allergen. (**H**) Effect of heat treatment on intrinsic fluorescence spectrum of *M. oleifera* leaf allergen. (**B**–**F**) Three technical replicates were conducted. Different superscript letters mean significant differences among different samples (*p* < 0.05).

Proteins exposed to high temperatures undergo denaturation, which is defined as a significant change in the natural three-dimensional conformation [23]. The side chain groups of amino acids are exposed in an unexpanded conformation during the heating process, which is usually conducive to denaturation [24]. Figure 5A shows the endothermic peak at 59.78 °C, which can be attributed to the thermal denaturation of the *M. oleifera* leaf allergen with a denaturation enthalpy of 61.4 J/g. This provides complementary evidence to that presented in Table 1.

### 2.7. Effect of Heat Treatment on Particle Size and Zeta Potential of M. oleifera Leaf Allergen

Higher protein concentrations increase their self-association capacity, resulting in an increase in the non-ideal properties of the solution [25]. As shown in Figure 5B, the ability of binding antibodies by proteins of a certain size of *M. oleifera* leaf allergen clusters decrease with an increasing heating temperature at pH 7. This may be due to the reduction in *M. oleifera* leaf allergen clusters after heat treatment and the more uniform dispersion of the *M. oleifera* leaf allergen in solution.

The zeta potential can reflect the surface charge state of particles in a dispersed system, thus influencing the surface charge [26]. It is also an important factor in determining the magnitude of electrostatic interactions between particles [27]. As shown in Figure 5B, the *M. oleifera* leaf allergen is negatively charged, and its electronegativity increases with an increasing temperature. The increase in electronegativity can be attributed to the exposure of charged amino acid residues on the protein surface.

### 2.8. Effect of Heat Treatment on Total Sulfhydryl (TSH) Content of M. oleifera Leaf Allergen

As shown in Figure 5C, the TSH content in the *M. oleifera* leaf allergen exhibits a decline with an increase in temperature. The observed decline in TSH content may be attributed to the formation of disulfide bonds and the establishment of connections with other amino acid residues during the heating process [28]. The findings indicate that elevated temperatures result in enhanced protein oxidation in the *M. oleifera* leaf allergen.

### 2.9. Effect of Heat Treatment on Surface Hydrophobicity of M. oleifera Leaf Allergen

As shown in Figure 5D, the surface hydrophobicity of the *M. oleifera* leaf allergen can be markedly enhanced through heat treatment. The hydrophobicity remains stable below 60 °C, decreases at 70 °C, and subsequently increases. Conversely, when the temperature is above 90 °C, the hydrophobicity increases significantly. Heat treatment can facilitate the exposure of hydrophobic amino acids to the surface. A protein with a higher surface hydrophobicity exhibits a lower solubility. The surface-exposed hydrophobic groups are involved in a wide range of intermolecular interactions, which ultimately results in a reduction in solubility.

### 2.10. Effect of Heat Treatment on Solubility of M. oleifera Leaf Allergen

The Bradford method was employed to assess alterations in the solubility of the *M. oleifera* leaf allergen. As shown in Figure 5E, the residual protein content in the solution following heating was compared with that of the untreated *M. oleifera* leaf allergen, with the assumption that the residual protein content was 100%. The findings revealed that the solubility of *M. oleifera* leaf allergen initially increased and then subsequently declined. This phenomenon may be attributed to the fact that temperatures below 70 °C facilitate the dissolution of the insoluble *M. oleifera* leaf allergen, resulting in a notable decline in solubility at temperatures exceeding 80 °C. The observed decline in solubility may be attributed to the aggregation and precipitation of the *M. oleifera* leaf allergen. According to the results of DSC, the protein may also be denatured at about 70 °C, resulting in a decrease in solubility.

### 2.11. Effect of Heat Treatment on Ultraviolet Spectrum (UV) of M. oleifera Leaf Allergen

As shown in Figure 5G, the UV absorption peak of the protein in the vicinity of 285 nanometers (nm) is attributed to the conjugated double bonds of the hydrophobic amino acids. The absorbance at all wavelengths detected in the figure increases with the temperature. The results of ultraviolet–visible spectroscopy indicate that as the temperature increases, there is exposure of more hydrophobic amino acid residues on the surface of the protein. Notwithstanding the presence of tyrosine residues within the molecule at elevated temperatures, exposure to other hydrophobic amino acid residues remained the dominant phenomenon.

### 2.12. Effect of Heat Treatment on Intrinsic Fluorescence Spectrum of M. oleifera Leaf Allergen

As shown in Figure 5H, the maximum fluorescence emission of the untreated *M. oleifera* leaf allergen was observed to occur at a wavelength of 327 nm. At a heating temperature of 40 °C, the fluorescent intensity reaches a maximum value, after which it decreases with the increase in temperature (50–100 °C). At 100 °C, the wavelength of the maximum emission is also observed to redshift to 332 nm. The observed changes in the maximum fluorescence intensity indicate that at lower heating temperatures, the *M. oleifera* leaf allergen unfolds gradually, resulting in the exposure of tyrosine residues to the solvent environment. However, at higher temperatures, the loss of fluorescence intensity may be attributed to protein aggregation. Furthermore, the redshift of the maximum emission wavelength suggests that the tertiary structure or aggregation of the protein may have undergone a change.

### 2.13. Effect of Heat Treatment on Protein Aggregates of M. oleifera Leaf Allergen

The fluorescence of Nile red binding to protein aggregates is specific and can be employed to evaluate protein aggregates [29]. In comparison to protein monomers, aggregates exhibit a greater affinity for Nile red, resulting in a higher fluorescence intensity. As shown in Figure 5F, the fluorescence content demonstrates a notable increase with elevated temperatures. Consequently, the *M. oleifera* leaf allergen aggregates during the heating process, with the formation of more aggregates at higher temperatures, which results in a structural alteration of the *M. oleifera* leaf allergen.

### 2.14. Effect of Heat Treatment on Simulated Digestibility In Vitro of M. oleifera Leaf Allergen

**Figure 6:**
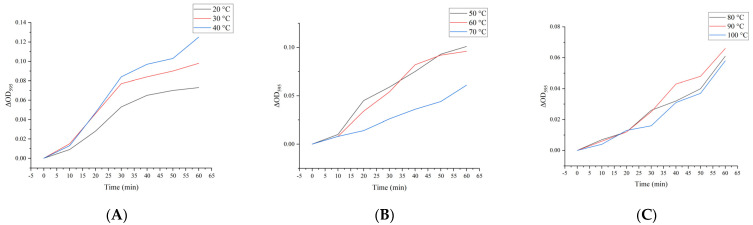
Effect of heating temperature on simulated gastric digestion of *M. oleifera* leaf allergen in vitro. (**A**) 20–40 °C; (**B**) 50–70 °C; (**C**) 80–100 °C.

As shown in Figure 6, the impact of temperature on gastric digestibility in *M. oleifera* leaf allergen samples is demonstrated. The gastric digestibility of the samples was found to be comparable when subjected to temperatures below 60 °C. The rate of gastric phase digestion is initially rapid but then gradually slows down after approximately 30 min (Figure 6A,B). As shown in Figure 6C, both the initial and final (60 min) digestion rates were negatively impacted by elevated temperatures. The application of a low-temperature treatment resulted in a notable enhancement in the digestibility of the samples, with gastric digestibility at 50 °C exhibiting an approximately 80% increase in comparison to the control group. However, an increase in temperature has been observed to inhibit the digestion of the *M. oleifera* leaf allergen.

**Figure 7:**
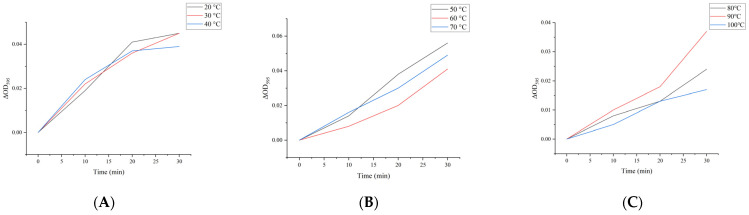
Effect of heating temperature on *M. oleifera* leaf allergen simulated intestinal digestion in vitro. (**A**) 20–40 °C; (**B**) 50–70 °C; (**C**) 80–100 °C.

The potential for protein degradation and sensitivity to trypsin are also affected by temperature, despite prior pepsin treatment. As shown in Figure 7, the intestinal digestibility of heat-treated samples at varying temperatures is presented.

### 2.15. Prediction Results of FBA Hydrophilicity

Following the completion of serum immunoblotting experiments, the predicted target was identified as a 36 kDa protein. We cut the bands corresponding to the molecular weight on the SDS-PAGE images, ESI/MS analyses, and gene retrieval experiments. In this study, we endeavored to make structural and functional predictions for *M. oleifera* FBA.

**Figure 8:**
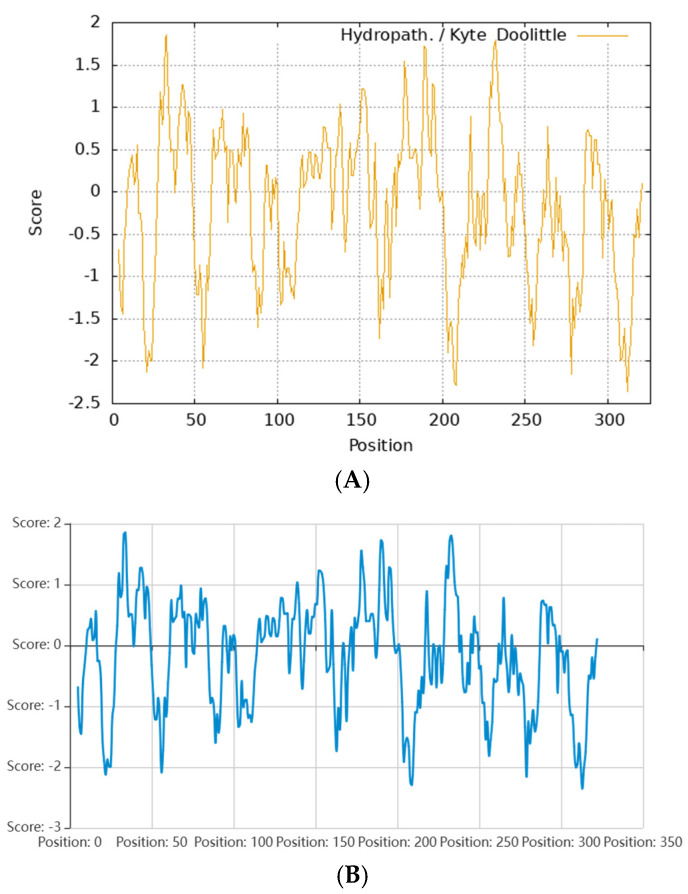
The hydrophilicity of FBA. (**A**) ExPASy; (**B**) Novopro. Hydrophobic is positive, hydrophilic is negative.

The hydrophilicity of FBA was analyzed using ExPASy-Prot Scale and Novopro. The results are presented in Figure 8A,B, respectively, where the region marked with a hyphen is the hydrophilic region. A comparison of the results obtained from two different websites revealed a high degree of consistency between the two methods. The amino acid sequence of FBA exhibited a wide range of hydrophilic regions, with the hydrophilic region being larger than the hydrophobic region, which occupied a dominant position. This indicates that FBA is a hydrophilic peptide.

### 2.16. Prediction of Transmembrane Region in FBA

**Figure 9:**
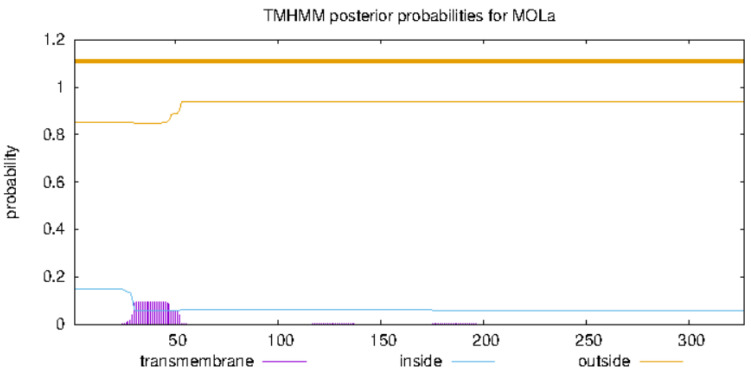
The transmembrane region of FBA.

The prediction of transmembrane protein structures is of great significance in the context of protein secondary structures, as well as in the growth and metabolism of organisms, internal and external signal transmissions, and energy conversion. As shown in Figure 9, the results indicated that the expected value of the number of helix amino acid residues in the protein was 2.04, which is less than the threshold value of 18. Therefore, FBA was found to lack a transmembrane domain, indicating that it is not a transmembrane protein and does not engage in transmembrane movement. This finding is consistent with the prediction regarding the hydrophilicity of these proteins.

### 2.17. Prediction Results of FBA Antigenicity Index

The parameters hydrophilicity, flexibility, accessibility, angle, exposed surface, polarity, and antigenicity of the polypeptide chain are related to the position of the continuous epitope [30]. This consequently gives rise to the pursuit of antigenic epitopes founded upon specific attributes of the protein sequence, along with the anticipation of the location of subsequent epitopes [31]. All prediction calculations are based on the relative propensity of amino acids to exhibit the properties described in the scale. The propensity scale comprises 20 amino acids, each with 20 values, which are ultimately assigned to each amino acid residue.

**Figure 10:**
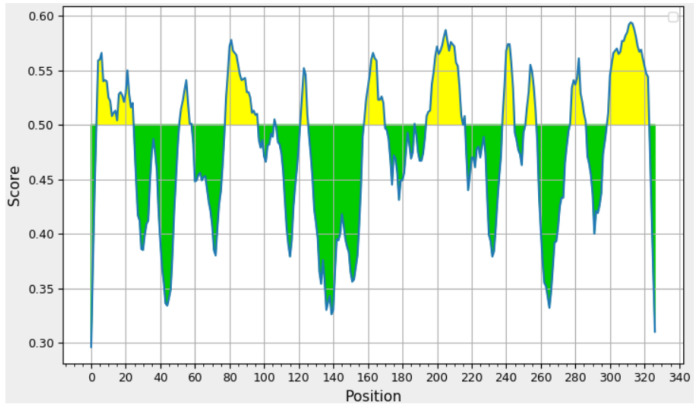
The antigenicity index of FBA. With 0.5 as the threshold, fragments greater than 0.5 are considered to have allergen activity and filled with yellow.

The FBA antigenicity index was subjected to analysis using IEDB, which employed the random forest algorithm to calculate epitopes and non-epitope amino acids determined from the crystal structure. Residues with scores exceeding the specified threshold (the default value is 0.5) are identified as potential epitope components and are represented in yellow on the plot. The plot presents the residue score on the y-axis and the residue’s position in the sequence on the x-axis. As shown in Figure 10, the results indicated that the antigen index of the majority of amino acid residues surpassed the default value of 0.5, with all of them exceeding 0.3. Ten peptide segments exhibited values exceeding the threshold value of 0.5, specifically AA4-24, AA51-58, AA78-96, AA121-125, AA158-169, and AA194-214. The remaining peptide segments are as follows: AA238-244, AA252-257, AA277-286, and AA299-322.

### 2.18. Prediction Results of FBA Calcium Binding Site

**Figure 11:**
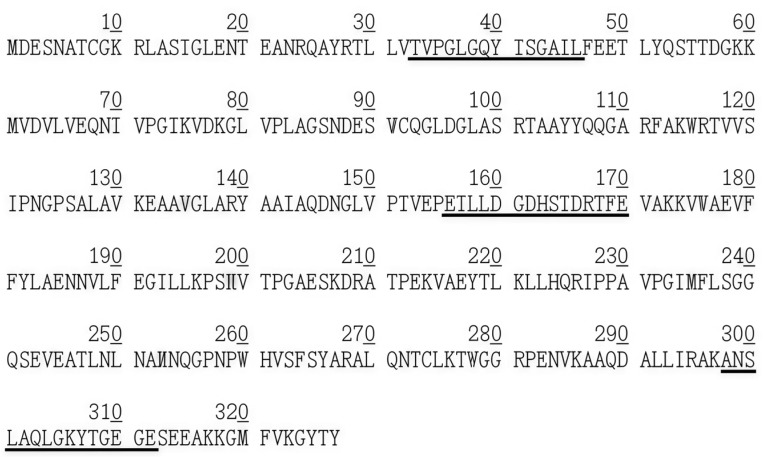
The predicted calcium binding site of FBA. The underlined sequences are predicted calcium binding sites.

As shown in Figure 11, the ExPASy-Peptide Cutter was employed to predict the calcium binding site of FBA. The results indicated that three peptides exhibited calcium-binding properties. The amino acid residues underlined in the figure are AA33-46, AA156-170, and AA298-312, respectively. It has been demonstrated that the sequence contains calcium-binding regions, which may act as sensitizers [32].

### 2.19. Prediction Results of FBA Secondary Structure

**Figure 12:**
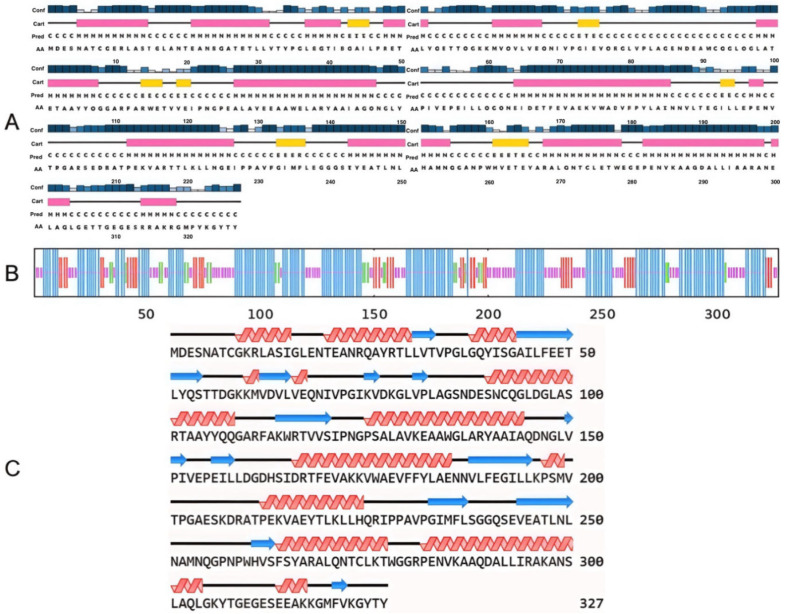
The secondary structure prediction of FBA. (**A**) The predicted result of PSIPRED, where pink is the α-helix, yellow is β-folding, and the gray line is random curling.Conf represents confidence. The higher the confidence, the darker the color (**B**) The predicted result of SOPMA, where blue is an alpha spiral, red is β-folding, green is a corner, and purple is random curling. (**C**) The predicted result of NovoPro, where the red spiral is the α-spiral, the blue arrow is the β-fold, and the black line is the random curl.

Three distinct methodologies were utilized to analyze the secondary structure of FBA. The software utilized for this analysis included PSIPRED, SOPMA, and NovoPro. Figure 12A illustrates the PSIPRED prediction, Figure 12B depicts the SOPMA prediction, and Figure 12C presents the NovoPro prediction. As shown in Figure 12, the results of the three methods indicate that the predominant secondary structure of FBA is an α-helix, followed by a random curling structure, an angle, and β-folding, collectively accounting for approximately half of the total secondary structure. Moreover, as shown in Table 2, the relative proportions of the diverse secondary structures within FBA were subjected to individual analysis. The PSIPRED and NovoPro analyses both indicated that the rotation angle was absent from the secondary structure of the protein. The results of the three methods indicated that FBA was a protein with a dominant α-helical secondary structure.

### 2.20. Prediction Results of FBA Three-Dimensional Structure

**Figure 13:**
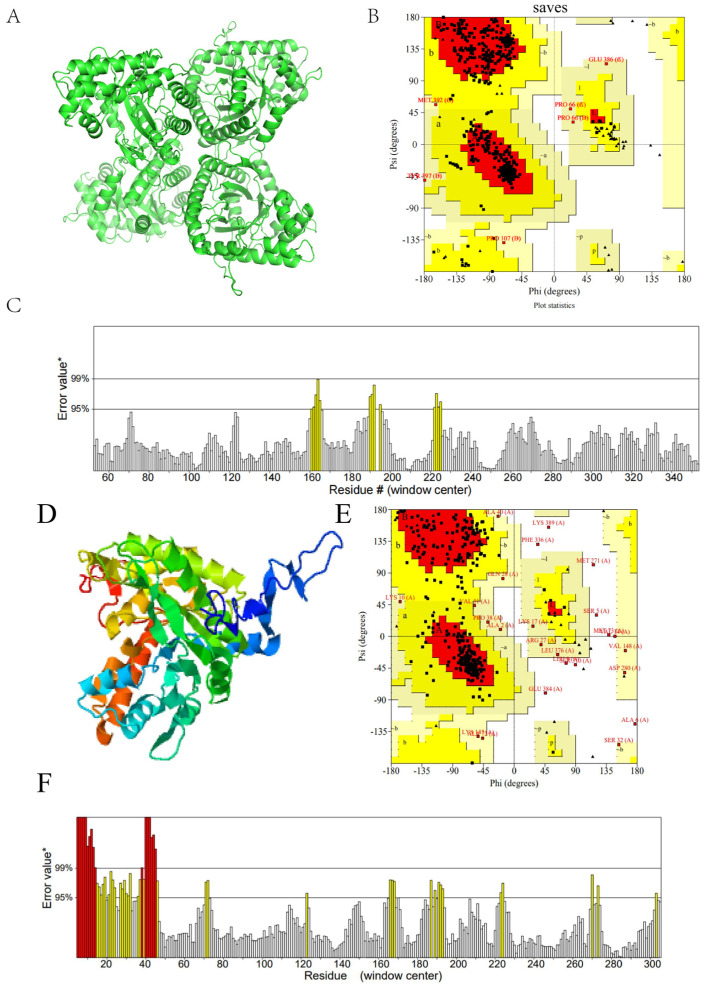
The three-dimensional structure prediction and quality analysis of FBA. (**A**) Swiss-Model prediction 3D model; (**B**,**C**) La diagram and resolution diagram of a; (**D**) I-TASSER prediction 3D model; (**E**,**F**) La diagram and resolution diagram of d. * On the error axis, two lines are drawn to indicate the confidence with which it is possible to reject regions that exceed that error value.

The Swiss-Model and I-TASSER online programs were utilized to predict the two three-dimensional structures of FBA. The two three-dimensional models are presented in Figure 13A,D. To assess their stereochemical quality, including ERRAT, the models were input into the SAVES_3 server, which also served to verify the 3D online tool. The evaluation of the FBA1 model is presented in Figure 13B,C. In the Lagerfeld diagram, 92.4% of the amino acid residues are in the most favored region, and no amino acid residues are in the disallowed region. The overall quality factor of the ERRAT2 figure is 95.545. The evaluation of the FBA2 model is presented in Figure 13E,F. The results indicate that 76.5% of the amino acid residues in the Lagree diagram are residues in the most favored region, while 2.9% of the amino acid residues are residues in the disallowed region. The overall quality factor of ERRAT 2 is 85.385.

A high-quality model is required for the Laplace diagram, with the expectation that more than 90% of the amino acid residues will be present in the most populated areas. ER-RAT2 represents a resolution structure. It is not uncommon for high-resolution structures to yield values of approximately 95% or more. For lower resolutions (2.5 to 3A), the mean overall quality factor is approximately 91%. In conclusion, FBA1 is selected as the 3D model for subsequent research based on the combined scores of both.

The α-helix is of pivotal importance in maintaining the relative stability of the spatial structure of protein molecules. The three-dimensional structure model (Figure 13A,B) illustrates the distribution of its secondary structure. In the entirety of the sequence, the secondary structure α-helix and random curling are the dominant forms, which aligns with the prediction of the *M. oleifera* leaf allergen secondary structure in Section 2.19. Furthermore, the results of the hydrophilicity analysis in Section 2.15. indicate that FBA exhibits high hydrophilicity. This assertion is supported by the observation that the spatial structure of FBA is not particularly complex and that the majority of amino acids are exposed to the exterior.

### 2.21. Conservative Analysis of FBA Three-Dimensional Structure

**Figure 14:**
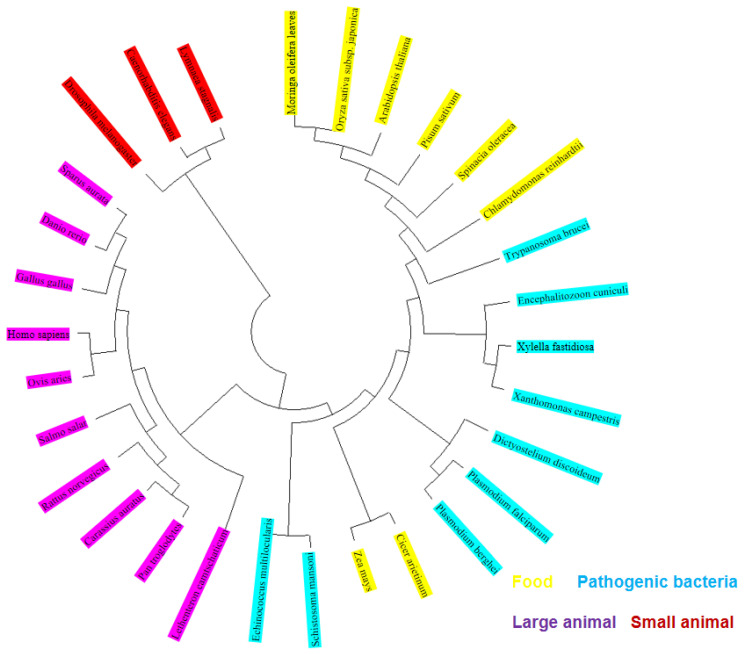
FBA phylogenetic tree was mapped using Bayesian and maximum likelihood methods.

**Figure 15:**
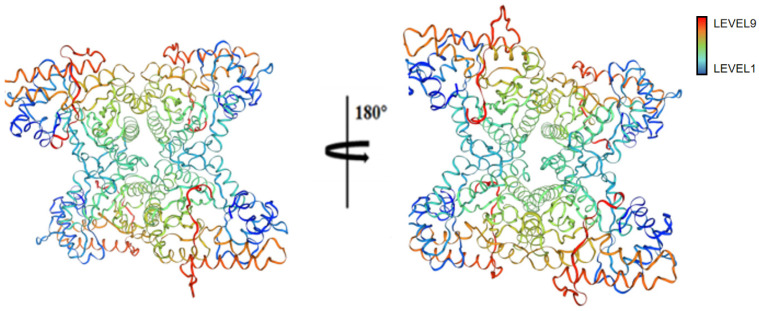
A single amino acid in FBA was subjected to a conserved analysis using the Consurf server. The color gradient, from blue to red, indicates a variable (level 1) to conservative (level 9) position.

The ConSurf online server was utilized to automatically identify FBA matches with 30 related species, and their homologous relationships were obtained. As shown in Figure 14, phylogenetic trees were constructed for comparative analysis. Furthermore, as shown in Figure 15, a conservative analysis of the FBA three-dimensional model was conducted.

The consensus trees generated for all proteomes exhibit comparable topologies and substantial branching support for FBAs of disparate species (Figure 14). The ConSurf identifier is mapped to the structure using the ConSurf color code, which indicates the level of structural protection. The colors blue, green, and yellow correspond to the variable (level 1) and position of protection (level 9), respectively. The color red indicates a more conservative position. As illustrated in Figure 15, the amino acid residues of FBA exhibit high levels of conservation, with notable differences concentrated in regions AA18-33, AA43-51, AA99-110, AA122-134, AA252-264, and AA332-334.

## 3. Discussion

*M. oleifera* is a rich source of nutrients and offers a multitude of benefits. The protein content of *M. oleifera* leaves is as high as 22% (dry weight), which is several times higher than the protein content of milk. It is particularly rich in high-quality protein [33]. However, during the promotion of *M. oleifera* leaves, it has been observed that some individuals may experience adverse reactions, including rashes, diarrhea, abdominal discomfort, dizziness, nausea, and vomiting, particularly when consuming fresh *M. oleifera* leaves. These adverse symptoms are considered to be allergic reactions, and our previous research has demonstrated that the consumption of spicy wood leaves can indeed result in the development of allergies in mice. The preliminary research conducted by the research group has revealed that the purified *M. oleifera* leaf allergenic protein is capable of activating dendritic cells in vitro and inducing CD4+ T cells to polarize toward the Th2 direction [34]. Additionally, *M. oleifera* leaf protein has been demonstrated to enhance intestinal permeability by impairing the integrity of the intestinal epithelial barrier through the TLR4 pathway [35]. The aforementioned evidence suggests that *M. oleifera* leaf protein exerts notable immunological effects and may, in certain instances, precipitate the development of allergic reactions. Zhang Jie and colleagues demonstrated that oral administration of *M. oleifera* leaf protein can induce allergic reactions in BALB/c mice [7]. Our findings align with those of other research groups. It is of great importance to study the allergenic activity of proteins and their immunomodulatory effects in order to prevent risks and promote the use of *M. oleifera* resources. Nevertheless, there is currently only a limited amount of research exploring the active proteins of *M. oleifera* leaves. The majority of current research is focused on the immunological effects of *M. oleifera* seed proteins and *M. oleifera* leaf whole proteins. Recently, the aforementioned studies have demonstrated the allergenicity of *M. oleifera* leaf protein. However, there have been no reports on related single-allergen information.

The experimental results demonstrated the presence of three principal proteins in *M. oleifera*, exhibiting molecular weights of 36 kDa, 45 kDa, and 55 kDa, respectively. The 36 kDa protein was identified by our research group, while the latter two were identified by Professor Huang Aixiang as serine/threonine endopeptidase and aspartate endopeptidase homologs. Of these, the 45 kDa protein has been demonstrated to possess lactase activity and has been the subject of extensive investigation by Wang Xuefeng’s research group [36].

It is not uncommon for plant proteins to elicit hypersensitivity reactions in the body. Indeed, protein allergies are a multifactorial disease. To reduce the allergenicity of allergens, a variety of methods are employed, including heat processing, such as stewing, combined cooking with ultrasound, high temperature and high pressure, and so forth. These methods are based on both the cooking methods used in everyday life and laboratory research methods. Zhang Fan and colleagues demonstrated that simulated stewing reduced the allergenicity of Pacific oyster allergens [37]. It is not the case that cooking can be employed as a universal solution to the problem of food allergies. In some instances, allergens that have been cooked have been observed to display increased allergenic activity or to produce other allergens. It is, therefore, of research value. Liu Guangming et al. found that heating obviously affects the biochemical characteristics of tropomyosin. The allergenicity of tropomyosin was decreased in high-temperature-pressure-processed crabs due to alteration in the protein structure [38]. Liu Meng and colleagues were able to significantly reduce the content and types of allergens present in the muscle of horseshoe crabs by subjecting them to high temperatures and high pressure [39]. Consequently, *M. oleifera* protein was subjected to diverse temperature treatments to emulate various cooking procedures. The findings of the research indicated that the protein present in *M. oleifera* leaves exhibited indications of aggregation accompanied by an increase in molecular weight and a reduction in soluble protein content. The IgE binding ability was found to decrease, while the hydrophobicity of the allergen surface increased following the simulated stewing of *M. oleifera* leaves. The average particle size and solubility decreased, which resulted in the aggregation of allergens and a change in allergen conformation. The findings of this experimental study provide guidance on the optimal cooking method for *M. oleifera* leaves.

The immunomodulatory effects of *M. oleifera* leaf protein have been the subject of considerable research in recent years. A comprehensive understanding of the key proteins that exert immune effects in *M. oleifera* leaf is essential for the optimal comprehension of the immune regulatory mechanism of *M. oleifera* leaf protein and the prevention of allergies. The application of bioinformatics can facilitate the analysis of the relationship between allergens and allergies, enabling the prediction of the function and structure of allergens. The 20 common amino acids that constitute proteins are classified as either hydrophobic or hydrophilic residues. The Hopp Woods antigen analysis, a classic approach in this field, suggests that hydrophilic residues are located on the surface of proteins, while hydrophobic residues are located inside the protein structure. The hydrophilic regions on the surface are conducive to the formation of antigen epitopes; however, highly hydrophilic regions are not necessarily antigen epitope regions. Further research is required to elucidate the consistency between the two and the coverage of the hydrophilic region by antigen epitopes. The results of the FBA hydrophilicity prediction indicate that the amino acid sequence contains a significant number of hydrophilic regions. Consequently, much of the sequence should be accessible to form a relatively extended structure, which is conducive to the formation of antigen epitopes and, thus, plays a sensitizing role. It is essential to predict the transmembrane region of a given sequence. The vast majority of potential antigen epitope sites are distributed throughout the entire sequence, indicating that FBA may possess multiple potential antigen epitope sites. These potential antigen epitopes also increase the likelihood of the protein itself being allergenic. It has been reported that the binding of Ca^2+^ is crucial for the formation of the conformational structure of calcium-binding proteins. Furthermore, the consumption of Ca^2+^ may lead to a decrease in IgE binding ability [40]. Further investigation is required to elucidate the impact of calcium on the structure and allergenicity of FBA. It is common for polypeptide chains to adopt a secondary structure comprising corners, which can then connect to other secondary structures, such as alpha helices and beta folds, within protein molecules. They are typically situated on the exterior of protein molecules and have the capacity to alter the orientation of polypeptide chains. Consequently, they are pervasively present in globulin. It is postulated by some researchers that the corners are predominantly protruding structures that manifest on the surface of antigens, which facilitates the formation of antigen epitopes that are readily chimeric with antibodies. However, regarding their respective structures and angles, the relatively stable alpha helix and beta fold are challenging to integrate. The hypothesis of antigen–antibody interaction posits that the assembly of difficult-to-assemble alpha helices and beta folds is an impediment to the formation of antigen epitopes. Therefore, an analysis based solely on the secondary structure of FBA does not allow us to conclude that it can form antigenic epitopes. This conclusion is at odds with the occurrence of sensitization phenomena in actual life and clinical practice. It is, therefore, recommended that the allergenicity of the protein be confirmed comprehensively by combining the secondary structure, amino acid sequence properties, and experimental verification. The spatial structure of FBA is conducive to the formation of antigen epitope sites. Furthermore, the combined effects of hydrophilicity, rotation angle, molecular polarity, and antigen index of the FBA amino acid sequence also determine its specific spatial structure. In light of the above, the structural prediction analysis results provide a theoretical basis for the sensitization of *M. oleifera* leaf allergens and also offer a certain foundation for a more comprehensive understanding and explanation of cross-reactivity. The secondary and tertiary structure predictions for FBA indicate that it is a conserved protein, being predominantly composed of alpha helices. Given the current lack of information regarding the three-dimensional structure and epitopes of FBA, further investigation is required to elucidate the impact of structural similarity on allergenicity. Accordingly, the conservative region analyzed in this study serves as a point of reference for subsequent research on the IgE binding epitope region of FBA and the cross-reactivity of FBA.

In conclusion, this study has successfully conducted a comprehensive analysis of FBA’s homology with other potential allergens. This has provided substantial data support for further studies into allergen cross-reactivity. The physicochemical properties and antigenic changes of *M. oleifera* leaf protein following heat treatment in solution were documented, and its physicochemical properties, antigenicity, and conservation were analyzed based on the sequence of FBA. These studies are of great significance for furthering our understanding of the sensitization mechanism of *M. oleifera* leaf protein and the relationship between *M. oleifera*leaf heat treatment and allergies. However, the samples used in the heat treatment are mixed protein samples, which may potentially impact the accuracy and reliability of the experimental results. As an extremely important functional protein and potential allergen in *M. oleifera* leaves, further investigation is warranted, particularly with regard to the biochemical properties of FBA. The findings of this study will contribute to our understanding of the immune regulatory mechanisms and properties of *M. oleifera* leaf proteins. The findings of this study will provide a framework for the assessment of the risk of *M. oleifera* allergy and facilitate further investigation into the immunomodulatory mechanisms of *M. oleifera* leaf protein.

## 4. Materials and Methods

### 4.1. Materials

The fresh *M. oleifera* leaves were purchased from Tianyou Technology Development Co., Ltd. (Dehong, Yunnan, China), transported on ice, and stored at −20 °C.

### 4.2. Preparation of M. oleifera Leaf Protein (M. oleifera Leaf Allergen)

First, 100 g of fresh *M. oleifera* leaves were weighed according to a material-to-liquid ratio of 1:6. Then, 600 mL of phosphate buffer salt solution (PBS) was added (Wuhan Servicebio Technology Co., Ltd., Wuhan, China). The *M. oleifera* leaves were juiced, *M. oleifera* leaf juice was placed at 4 °C, filtered, with the filter residue discarded, and centrifuged at 4000 rpm for 30 min, after which the residue was removed and the supernatant collected. Ammonium sulfate (Chongqing Chuandong Chemical (Group) Co., Ltd., Chongqing, China) was added to the supernatant until 60% saturation, then stirred evenly, left for 7 h, and centrifuged at 4000 rpm for 30 min, after which the supernatant was removed, the precipitate was collected, the precipitate was dissolved with PBS, the solution was put into the dialysis bag (Shanghai Yuanye Bio-Technology Co., Ltd., Shanghai, China), and the dialysis bag was put into the ultrapure water bucket, where it was stirred and desalted until the ammonium sulfate was removed. The samples were lyophilized at −50 °C. The obtained samples were stored at −20 °C.

### 4.3. Determination of the Hemagglutination Titer

Rabbit blood (Defibrinated rabbit blood from Beijing Solarbio Technology Co., Ltd., Beijing, China) was mixed with anticoagulant (Aldrin solution preparation: glucose 2.05 g, sodium citrate 0.8 g, citric acid 0.055 g, sodium chloride 0.42 g, and distilled water to 100 mL; pH to 6.1; sterilized at 115 °C in autoclave for 10 min; stored at 4 °C) at [V(blood):V(anticoagulant) = 1:9]. Slowly, the blood collection tube was inverted so that it was thoroughly mixed and stored at 4 °C for later use. It was washed with 0.9% normal saline, repeated 4–5 times (centrifuge at 1800 rpm for 10 min) until the supernatant was transparent and clear; then, the supernatant was discarded, 0.9% normal saline (Zhejiang Tianrui Pharmaceutical Co., Ltd., Zhejiang, China) was added to prepare a 2% (*v*/*v*) red blood cell suspension, and the prepared red blood cell suspension was stored at 4 °C.

To a V-type hemagglutination plate, we added 25 μL of 0.09% normal saline into each well. Then, 25 μL of sample to the first well of each row. After mixing well, 25 μL was aspirated from the first well and moved to the second well. After mixing, 25 μL was aspirated and moved to the third well. Then, 25 μL of 1% rabbit erythrocyte suspension was added to each hole, and the V-type hemagglutination plate was oscillated on a micro-oscillator for 1 min to mix completely, and the hemagglutination result was observed via the naked eye after 2 h at room temperature, expressed by 2^n^ (^n^ is the number of holes). According to the hemagglutination test results, if all groups of controls are correct, the hemagglutination reaction intensity is determined according to the following criteria: titer determination: significant agglutination, “++++”; most agglutination, “+++”; some agglutination, “++“; little agglutination, “+”; and no agglutination: “-”.

### 4.4. Heat Treatment

The lyophilized samples were dissolved in PBS (4 mg/mL). A 10 mL solution was heated in a water bath at 30, 40, 50, 60, 70, 80, 90, and 100 °C for 20 min to simulate braising (water heating). The center temperature of the sample was measured using a temperature recorder (MIK-R200D, Hangzhou Asmik Sensors Technology Co., Ltd., Hangzhou, China). The heated solution was cooled at room temperature (20–25 °C) and shaken for further analysis. The samples were stored at −20 °C.

### 4.5. SDS-PAGE

A 12% Separation Gel and 4% Concentration Gel were used. Approximately 20 μL of the sample solution was mixed with 5 μL of 5 × sample buffer (Beyotime Biotechnology Co., Ltd., Shanghai, China). The mixture was boiled at 100 °C for 10 min, and then 20 μL of each cooled mixture was loaded onto the gel. The gel was run on a Mini-Protean III system (Bio-Rad Laboratories Co., Ltd., Hercules, CA, USA). After electrophoresis, the sample was stained with Coomassie brilliant blue R-250 (Beijing Chemical Industry Group Co., Ltd., Beijing, China) (dissolved in 45% deionized water, 45% methanol, and 10% acetic acid) for 1 h and decolorized with decolorization solution (10% methanol, 10% acetic acid, and 80% deionized water) overnight until completely decolorized.

### 4.6. Differential Scanning Calorimetry (DSC)

The denaturation temperature of the sample was measured using DSC (DSC 200 PC, NETZSCH-Gerätebau GmbH Co., Ltd., Gebrüder-Netzsch-Straße 19, Selb, Germany). The 10–20 mg freeze-dried sample was placed in an aluminum dish and sealed, while an empty dish was used as a reference. The sample was heated from 25 to 105 °C at 10 °C /min. These experiments were performed at least twice. The inflection point of the DSC curve is the thermal transition temperature of the sample, and the corresponding area between the air baseline and the sample curve is the enthalpy of protein denaturation, as calculated by the instrument software.

### 4.7. Particle Size and Zeta Potential

The particle size and zeta potential of the sample were determined using a particle size analyzer (Zetasizer Nano ZS90, Malvern Panalytical Co., Ltd., Malvern, UK) Here, 2 mg/mL heated sample solution was adjusted to pH 7 with 1 M hydrochloric acid and 1 M sodium hydroxide. Then, ~1.5 mL of sample solution was added to the static sample pool (1 cm path length) and placed at 25 °C for 2 min. The zeta potential of the sample was determined using DTS1070 folded capillary cells (Malvern Panalytical Co., Ltd., Malvern, UK), repeated 3 times, and the mean value was reported. The mean particle size was measured using dynamic light scattering (DLS), which gives the hydrodynamic diameter of the OWSP cluster by measuring the diffusion coefficient of Fick’s first/second law and converting it to the mean particle size [41], repeated 3 times, and the mean value was reported.

### 4.8. Total Sulfhydryl (TSH) Content

According to a previous report [42], the TSH content was determined. The concentration of the sample solution was adjusted to 2 mg/mL with PBS. Then, 1 mL of sample solution was mixed with 3 mL of 0.2 M Tris-HCl buffer (8 M urea, 10 mM EDTA, and 2% SDS; pH 6.8) and 0.4 mL of Ellman reagent (0.1% DTNB and 0.2 M Tris-HCl; pH 8). The mixture was incubated at 40 °C for 25 min and then centrifuged at 6000 rpm at 4 °C for 10 min. The absorbance of the supernatant was measured at 412 nm using an ultraviolet–visible spectrophotometer (UV-VIS) (SMA4000, Merinton Co., Ltd., Beijing, China). The molar extinction coefficient of 1.36 × 10^4^ M/cm was used to calculate the TSH content, expressed as mol/10^5^ g crude protein [43]. The protein content of the samples was determined using the BCA Protein Assay Kit (Labgic Technology Co., Ltd., Beijing, China). This was repeated 3 times, and the average was reported.

### 4.9. Protein Solubility

The solubility of the samples was determined using the Bradford method. The protein standard solution was prepared with 100 μL BSA solution at concentrations of 0.0, 0.1, 0.2, 0.4, 0.6, 0.8, and 1.0 mg/mL. The Bradford working fluid was prepared by weighing 0.05 g of G250 into a beaker, adding 25 mL of 95% ethanol, stirring and dissolving on a magnetic stirrer at 400 rpm, then adding 60 mL of 85% phosphoric acid solution, and finally making up the solution to 500 mL with ultrapure water. It was filtered using Whatman L filter paper (General Electric Company Co., Ltd., Shanghai, China). Then, 30 μL of standard solution of different protein concentrations was added to 1 mL of Bradford working solution, gently inverted and mixed, and allowed to stand for 5 min. After diluting the 4 mg/mL sample 10 times, 30 μL was added to 1 mL of Bradford working solution, gently inverted and mixed, and left to rest for 5 min. The reaction solution at a volume of 200 μL was added to the sample well of the 96-well plate, and the absorbance at 595 nm was measured with an enzyme-labeled instrument (Flestation 3, Molecular Devices Co., Ltd., San Jose, CA, USA). The protein concentration of the sample was calculated from the standard curve. The solubility of the sample was determined by measuring the residual protein content of the treated solution.

### 4.10. Surface Hydrophobic

According to a previous report [44], the surface hydrophobicity was determined. The concentration of the sample solution was adjusted to 2 mg/mL with PBS (pH 6). A total of 80 μL of 1 mg/mL BPB (in deionized water) was added to 2 mL of the sample, and the solution was stirred at 200 rpm for 10 min at room temperature. The absorbance of the supernatant was measured at 595 nm via centrifugation at 2000 rpm for 10 min, and 2 mL of PBS of the same treatment was used as a control. The formula for calculating the amount of BPB binding was as follows:

$$\mathrm{Bound}\;\mathrm{BPB}\left(\mu g/\mathrm{mg}\;\mathrm{MOL}\right)=\frac{80\mu g\times \left(\mathrm{OD}\;\mathrm{control}-\mathrm{OD}\;\mathrm{sample}\right)}{4\;\mathrm{mg}\;\mathrm{MOL}\;\mathrm{OD}\;\mathrm{control}}$$

### 4.11. Intrinsic Fluorescence Spectrum

The intrinsic fluorescence spectra of the sample solution were measured using a fluorescence spectrophotometer (F-7000, Hitachi Co., Ltd., Tokyo, Japan). The sample solution (2 mg/mL) was diluted and heated 3 times with PBS (pH 7). Then, 3 mL of each diluted sample was added to a quartz dish with a path length of 1 cm and excited at 295 nm (both the excitation and emission slit widths were 5 nm). The emission spectrum measurement range is 300~400 nm, and the scanning speed is 5 nm/s.

### 4.12. Ultraviolet Spectrum

Ultraviolet–visible spectra were measured with a UV-VIS instrument (SMA4000, Merinton Co., Ltd., Mason, OH, USA) at room temperature with a 1 cm sample solution (0.5 mg/mL). The scanning range is 225~450 nm, and the scanning speed is 5 nm/s.

### 4.13. Protein Aggregates

Protein aggregates were detected using the fluorescence method. The sample concentration was adjusted to 1 mg/mL with PBS (pH 6), and then 30 μL of Nile Red solution (0.32 mg, 1 mL, 100% ethanol) (Shanghai Aladdin Biochemical Technology Co., Ltd., Shanghai, China) was added to 3 mL of sample solution. After shaking, 3 mL of each sample was added to a 1 cm path length quartz cell and measured at the optimal excitation and emission wavelengths (λex = 560 nm and λem = 620 nm, with both excitation and emission slit widths of 5 nm). The low autofluorescence of the sample was subtracted from the sample without Nile Red. Fluorescence intensity is expressed in arbitrary units (au). This was repeated 3 times, and the average was reported.

### 4.14. In Vitro Digestibility

#### 4.14.1. In Vitro Pepsin Digestion

The in vitro enzymatic digestibility of the protein was determined. The sample was dissolved in 33 mM glycine buffer at pH 2, and the final concentration was adjusted to 1 mg/mL. Pepsin at a concentration of 10 U/mg was added, and the protein underwent enzymolysis at 37 °C for 90 min. The volume of the reaction system was 100 mL, 10 mL was removed at different times (0, 10, 20, 30, 40, 50, and 60 min), and then 15% (final concentration) TCA (Beijing Solarbio Science & Technology Co., Ltd., Beijing, China) was added to stop the digestion. After centrifugation at 12,000 rpm for 2 min at 4 °C, the absorbance at 595 nm was determined using the Bradford method, and the protein content in the supernatant was calculated. Gastric digestibility was calculated as follows:

ΔOD595=ΔOD595 sample−ΔOD595 0 min

#### 4.14.2. In Vitro Trypsin Digestion

Pepsin activity was inhibited by adjusting the pH to 7.5 with 1 M hydrochloric acid and 1 M sodium hydroxide. Pancreatic protein at a concentration of 6.6 U/mg was added and hydrolyzed at 37 °C for 30 min. The volume of the reaction system was 100 mL, 10 mL was removed at different times (0, 10, 20, and 30 min), and then 15% (final concentration) TCA was added to stop the digestion. After centrifugation at 12,000 rpm for 2 min at 4 °C, intestinal digestibility was determined as described above.

### 4.15. Prediction of FBA Hydrophilicity

ExPASy ProtScale (https://web.expasy.org/protscale/ (accessed on 20 September 2022)) and Novopro (https://www.novopro.cn/tools/protein-hydrophilicity-plot.html (accessed on 21 September 2022)) were used to analyze the hydrophilicity of FBA, and the results of both were compared.

### 4.16. Prediction of FBA Transmembrane Region

TMHMM-2.0 (https://services.healthtech.dtu.dk/services/TMHMM-2.0/ (accessed on 16 October 2022)) was used to analyze the FBA transmembrane region.

### 4.17. Prediction of FBA Antigenicity Index

The IEDB index application (http://tools.iedb.org/bcell/ (accessed on 18 October 2022)) was used to analyze the FBA antigenicity index.

### 4.18. Prediction of FBA Calcium Binding Site

ExPASy-Peptide Cutter (https://www.expasy.org/resources/peptidecutter (accessed on 22 October 2022)) was used to analyze the FBA calcium binding site.

### 4.19. Prediction of FBA Secondary Structure

Based on the amino acid sequence of FBA, PSIPRED (http://bioinf.cs.ucl.ac.uk/psipred/ (accessed on 29 October 2022)), NovoPro (https://www.novopro.cn/tools/secondary-structure-prediction.html (accessed on 29 October 2022)), and SOPMA (https://npsa-pbil.ibcp.fr/ (accessed on 29 October 2022)) were used to analyze the FBA secondary structure.

### 4.20. Prediction of FBA Three-Dimensional Structure

I-TASSER (https://zhanggroup.org/I-TASSER/ (accessed on 3 November 2022)) and SWISS-MODEL (https://swissmodel.expasy.org/interactive (accessed on 5 November 2022)) were used to analyze the FBA three-dimensional structure. SAVES (https://saves.mbi.ucla.edu/ (accessed on 8 November 2022)) was used to evaluate quality of stereochemistry of the model. ConSurf (https://consurf.tau.ac.il/ (accessed on 10 November 2022)) was used to analysis the conservatism of the FBA three-dimensional structure. FBA was compared with the FBA amino acid sequences of 30 related species, and the conservative analysis of the FBA three-dimensional model was obtained.

### 4.21. Immunoblotting

The protein was transferred to a 0.45 μm PVDF membrane (Millpore, Darmstadt, Germany) using the wet transfer method. The transfer conditions were set to a constant 240 mA and 65 min. It was sealed with 5% BSA (Biosharp, LABGIC Technology Co., Ltd., Beijing, China) using a solution prepared with TBST (10 × TBST: 121.1 g Tris base, 87.66 g NaCl, and 10 mL Tween 20; pH 7.6; volume adjusted to 1 L with ultrapure water). The first antibody was incubated with allergic mouse serum at 4 °C and 30 rpm in a shaker bed overnight. The second antibody was incubated with HRP-labeled IgE (Beijing Solarbio Technology Co., Ltd., Beijing, China) at room temperature for 1 h at 20 rpm.

Allergic mouse serum primary antibody: According to the proportion of serum (5% sealing solution = 1:100), 100 μL of allergic mouse serum was added to 10 mL of sealing solution, diluted and mixed, and then stored in a refrigerator at 4 °C.

Hunan Slakejing Da Laboratory Animal Co., Ltd. (Changsha, China) provided male BALB/c and C57 mice (5–6 weeks old, SPF) maintained on an allergen-free diet under pathogen-free conditions. These mice were maintained in an animal room with a temperature of 23 ± 3 °C and humidity of 50 ± 10%, and an alternating 12:12 h light–dark cycle, all under SPF conditions.

### 4.22. Data Analysis

All data in this study were derived from the mean of three or more independent experiments and expressed using the standard deviation (SEM). GraphPad Prism 5.01 was used to analyze the data, and one-way ANOVA was used to conduct comparisons between groups. * *p* < 0.05 indicates a statistically significant difference.

## 5. Conclusions

In summary, this experiment preliminarily investigated the lectin activity and allergenic activity of *Moringa oleifera* leaf protein and tested the effects of different temperature treatments on the physicochemical properties and lectin activity of *Moringa oleifera* leaf protein. According to the results of our experiment, 36 kd was inferred to be the major allergen in *Moringa oleifera* leaf protein. According to the database search, it was found to be FBA, and its protein properties were predicted. The experimental material is a mixed protein, and the effect of heat treatment was consistent with the actual protein state during production. However, the experiment cannot represent the properties of a single protein, and more in-depth research needs to be conducted after the extraction of the monomer.

## Figures and Tables

**Table 1:** Hemagglutination titer of *M. oleifera* leaf allergen with heat treatment. “+” stands for positive, and the more the number, the stronger the positive; “-” stands for negative.

Temperature	Hemagglutination Titer/2^n^								
	2^1^	2^2^	2^3^	2^4^	2^5^	2^6^	2^7^	2^8^	2^9^
Control	++++	++++	+++	++	++	+	+	-	-
30 °C	+++	+++	+++	++	++	+	+	-	-
40 °C	+++	+++	++	++	+	+	-	-	-
50 °C	+++	+++	++	++	+	+	-	-	-
60 °C	++	++	+	+	-	-	-	-	-
70 °C	+	+	-	-	-	-	-	-	-
80 °C	+	-	-	-	-	-	-	-	-
90 °C	-	-	-	-	-	-	-	-	-
100 °C	-	-	-	-	-	-	-	-	-

**Table 2:** Results of the secondary structure analysis using three different methods.

Method	α-Helix	β-Sheet	β-Turn	Random Coil
PSIPRED	47.4% (155)	6.7% (22)		45.9% (150)
SOPMA	51.7% (169)	12.8% (42)	7.3% (24)	28.1% (92)
NovoPro	44.0% (144)	18.3% (60)		37.6% (123)
Mean	47.7%	12.6% (60)	3.2%	37.2% (123)

## Data Availability

The original contributions presented in the study are included in the article; further inquiries can be directed to the corresponding authors.
